# A Three-Dimensional Dense Collagen Hydrogel to Model Cancer Cell/Osteoblast Interactions

**DOI:** 10.3390/jfb9040072

**Published:** 2018-12-12

**Authors:** Mark James-Bhasin, Peter M. Siegel, Showan N. Nazhat

**Affiliations:** 1 Department of Mining and Materials Engineering, McGill University, Montréal, QC H3A 0C5, Canada; mark.james-bhasin@mail.mcgill.ca; 2 Departments of Medicine, Biochemistry and Anatomy & Cell Biology, McGill University, Montréal, QC H3A 0C7, Canada; peter.siegel@mcgill.ca; 3 Rosalind and Morris Goodman Cancer Research Centre, McGill University, Montréal, QC H3A 1A3, Canada

**Keywords:** breast cancer, osteoblasts, bone metastasis, dense collagen hydrogel, mineralization

## Abstract

No curative treatment options exist once breast cancer metastasizes to bone. This is due, in part, to an incomplete understanding of how osteolytic cancers interact with bone. Presented here is a novel approach to study the interactions between triple negative breast cancer cells and osteoblasts within a 3D collagenous environment. More specifically, a dense collagen hydrogel was employed to model interactions between MDA-MB-231 breast cancer cells and MC3T3-E1 pre-osteoblasts. Co-cultures with these two cell types, or MDA-MB-231-derived conditioned medium applied to MC3T3-E1 cells, were established in the context of plastically compressed dense collagen gel matrices. Importantly, breast cancer-derived conditioned medium or the establishment of breast cancer/osteoblast co-cultures did not negatively influence MC3T3-E1 cell viability. The inclusion of either conditioned medium or the presence of MDA-MB-231 cells resulted in impaired MC3T3-E1 differentiation into osteoblasts, which coincided with reduced osteoblast-mediated mineralization. The results presented here demonstrate that dense collagen gels provide a model environment to examine the effect of osteolytic breast cancer cells on osteoblast differentiation and subsequent mineralization of the collagen scaffold.

## 1. Introduction

The “seed and soil” hypothesis first described by Stephen Paget still governs our view of cancer metastasis [1]. The central tenant of this hypothesis is that the metastatic cancer cells must possess a compatibility with the microenvironment to enable successful colonization and the formation of metastases [2,3]. Breast cancers can be sub-divided into several intrinsic subtypes, which include Luminal A, Luminal B, human epidermal growth factor receptor 2 (HER2+), and basal breast cancer, and show a high propensity to spread to bone [4]. Luminal A breast cancer spreads primarily to the bone only, whereas Luminal B, HER2+, and basal breast cancers metastasize to bone and soft tissue sites. Basal breast cancers, which include triple negative breast cancer (TNBC), are a particularly poor-prognosis subtype, and limited treatment responses and relapse are of grave clinical concern [5,6]. Progression to palliative care and subsequent morbidity are associated with a patient diagnosis of metastatic osteolytic TNBC [7,8].

Osteolytic TNBC results in the loss of bone mineral density [9]. Bisphosphonate therapy is used clinically as it prevents osteoclast-mediated bone resorption and alleviates skeletal-related events (fracture, pain) in patients with bone metastatic breast cancer [10]. Loss of bone mineral density can result from increased osteoclast-mediated bone resorption or suppression of osteoblast differentiation and new bone formation. We have gained a considerable understanding of how cancer cells can influence osteoclast and osteoblast functions during metastasis; however, considerably less is known regarding how breast cancer cells alter osteoblast-mediated mineralization.

Tissue engineering approaches that seek to recapitulate clinical phenomena have been guided by a need to understand disease etiology and progression from alternative systems using 3D scaffold environments [11,12,13]. Developing such environments would permit the dissection of heterotypic interactions between TNBC cells and osteoblasts in a microenvironment that more closely resembles bone. A plastically compressed, dense collagen (DC) gel system has been shown to dually support both seeded cell growth, and to provide a highly potent osteoid-like environment for the nucleation and growth of crystalline hydroxyapatite (HA) [14,15,16,17,18,19]. In contrast to other in vitro model systems [20,21,22,23,24], DC scaffolds allow pre-seeding of cells into 3D randomly-organized nanofibrillar gel matrices, prior to compression and fabrication of the final scaffold [14]. Most importantly, it allows for reductionist-level experimental analyses to examine interactions between cancer cells and bone cells in the correct matrix context. Indeed, matrix components found in preparations, such as Matrigel^®^ (e.g., laminins), which are not typically present in bone, are eliminated in favor of a collagen-based environment [18,25]. This provides a means to model mineralization within a system that includes only cells, and cell-derived factors; reducing the variables that are present in the in vitro environment.

The aim of this study was to develop 3D cultures of pre-osteoblasts and TNBC cells in DC constructs in order to: (1) assess the impact of a highly bone metastatic subpopulation of MDA-MB-231 breast cancer cells on the proliferation and differentiation of pre-osteoblasts in co-cultures, and (2) compare the impact of either co-cultures or MDA-MB-231-derived conditioned medium (CM) on osteoblast-mediated mineralization.

## 2. Materials and Methods

### 2.1. Cell Culture

A subpopulation of the MDA-MB-231 cell line that is highly metastatic to bone (1833-TR) has been described previously [26]. Breast cancer cells were grown in Dulbecco’s Modified Eagle’s Medium (DMEM, Wisent, St-Bruno, QC, Canada), 1% (v/v) non-essential amino acids, 1% (v/v) penicillin–streptomycin (Life Technologies, Thermo Fisher Scientific, Walthan, MA, USA).

MC3T3-E1 C57BL/6 mouse pre-osteoblast cells (subclone 14) were obtained from the ATCC (CRL2594, Manassas, VA, USA). Basal medium for MC3T3-E1 cells included Minimal Essential Eagle’s Media (MEM; Wisent, St-Bruno, QC, Canada) with 0.5% (v/v) non-essential amino acids, 0.5% (v/v) essential amino acids, 100 mg/mL ᴅ-calcium pantothenate, 100 mg/mL choline chloride, 100 mg/mL folic acid, 200 mg/mL i-inositol, 100 mg/mL nicotinamide, 100 mg/mL pyridoxine HCl, 10 mg/mL riboflavin, and 100 mg/mL thiamine HCl, to mimic alpha-modified MEM without ascorbic acid. Osteogenic-supplemented medium consisted of alpha-modified MEM (Alpha-MEM; Wisent, St-Bruno, QC, Canada) with the addition of (+)-sodium ʟ-ascorbate (Sigma-Aldrich, Oakville, ON, Canada) to a final concentration of 50 µM, and β-glycerophosphate disodium salt hydrate (Sigma-Aldrich, Canada) to a final concentration of 10 mM. CM consisted of MC3T3-E1 basal growth media with 5% (v/v) supplementation of medium harvested from cultures of 1833-TR cells. CM was first harvested after 48 h, the media replenished, and a second harvest was taken 48 h later. The CM was subsequently filtered twice with a 200 nm nylon syringe filter (BD Biosciences, Canada). All media used was complete by addition of 10% (v/v) fetal bovine serum (FBS, Thermo-Fisher, Walthan, MA, USA).

### 2.2. Construct Preparation and Cellularization 

As previously described [14], a four in five dilution of 10× DMEM (Sigma-Aldrich, Oakville, ON, Canada) was added to 0.6% acetic acid-solubilized rat tail tendon-derived type I collagen (2.1 mg/mL, First Link, Birmingham, UK). Subsequently, the collagen mixture was neutralized by the addition of 19 μL/mL 5 N NaOH to reach physiologic pH. 1833-TR and MC3T3-E1 cells were counted and seeded into the neutralized collagen mixture as follows: 2 × 10^5^ MC3T3-E1 cells alone, 1 × 10^5^ 1833-TR cells alone, or both cell lines together (at their respective seeding densities). 1833-TR cells were also plated in routine tissue culture flasks for CM preparation. Cell-seeded collagen underwent a process of gellification in 24-cell culture plates at 37 °C in a reducing atmosphere (5% CO_2_) for 25 min. The resulting highly-hydrated collagen gels were then transferred to the compression apparatus [15], and an unconfined compressive stress of 2.77 kPa was applied for 300 s to generate DC gels of 6.07 ± 1.82 wt % fibrillar collagen.

### 2.3. Assessment of Cell-Seeded Constructs with Time in Culture

#### 2.3.1. Confocal Laser Scanning Microscopy (CLSM)

CLSM (LSM510 Zeiss, Dorval, QC, Canada) was used to assess cell viability and morphology at days 7, 15, and 21 post-seeding. Constructs were washed in phosphate buffered saline (PBS) for 2 min and stained with 0.1 M calcein AM and 0.1 M Hoechst 33342 (Life Technologies, Thermo Fisher Scientific, Walthan, MA, USA). Excitation occurred with an argon laser (near λ_EX_ = 490 nm) and UV source (near λ_EX_ = 360 nm), both filtered (where λ_EM_ > 530 nm) for fluorescence/emission. Data collection occurred for 15 equidistant intervals in z-stacks (45 µm z-plane) with Fiji software (ImageJ; NIH, Bethesda, MD, USA) superimposed maximum intensity for display [27].

#### 2.3.2. Scanning Electron Microscopy (SEM)

SEM of 5% glutaraldehyde fixed, contrast-enhanced (0.01 M OsO_4_), constructs was carried out by successive ethanol washes (3 × 15 min each) in a gradient of 5%, 55% and 95% ethanol to displace the aqueous medium. Constructs were then incubated in anhydrous ethanol overnight. Bis(trimethylsilyl)amine was used to further displace ethanol, which was then removed by dry air under ambient conditions. Sputter coating was then applied as follows: 28 s of 90% gold/10% palladium mixture, after which the samples were degassed for 24 h. Analysis was carried out on a JEOL JSM-7400 FE-SEM (JEOL, Tokyo, Japan) with the secondary electron detector at 1.5 kV and 20 A.

#### 2.3.3. Resorufin Quantification Assay

A commercial resazurin-reduction assay (alamarBlue^®^, Life Technologies, Thermo Fisher Scientific, Walthan, MA, USA) was used to analyze the metabolic activity of the seeded cells up to day 24 in culture, as described previously [28]. Briefly, a 10% (v/v) alamarBlue^®^ solution with 5% (v/v) FBS supplemented basal medium and 1% (v/v) penicillin–streptomycin was used to replace the culture media. Triplicate samples were incubated for 2.5 h (protected from light) in a humidity-controlled 37 °C sterile incubator at 5% CO_2_, and agitated every 15 min. Samples were replaced with the respective media. Aspirates from the alamarBlue^®^ incubation were then measured using a TECAN^®^ 9600 (TECAN, Männedorf, Switzerland) epi-fluorescent plate reader (λ_EX_ = 535 nm and λ_EM_ = 600 nm).

#### 2.3.4. Cell-Mediated Gel Contractility Assay

A cell-mediated gel contractility assay was performed using a previously described method [29], and the surface area change of triplicate DC constructs was quantified from images generated at 600 dpi in 2D on a flatbed document scanner. Fiji measurements of the surface area change were taken every 24 h up to day 21 in culture.

#### 2.3.5. Quantitative RT-PCR (qPCR)

qPCR was performed on RNA isolated from whole collagen constructs. Briefly, homogenization of the cellularized collagen scaffold was performed, and the resulting lysate was loaded onto PureLink^®^ RNA mini kit columns (Life Technologies, Thermo Fisher Scientific, Walthan, MA, USA) for RNA extraction. Complementary DNA (cDNA) synthesis for reverse transcription was conducted using 500 ng of total RNA in RNAlater^®^ (Life Technologies, Thermo Fisher Scientific, Walthan, MA, USA): 200 U/µL M-MulV reverse transcriptase (New England Biolabs, Ipswich, MA, USA), 2.5 mM of each deoxynucleotide triphosphate, oligo-d(T) at 50 µM, RNase inhibitor at 10 U/µL, a 10× reaction buffer. Reactions were brought to a total volume of 50 µL and purity was verified by spectrophotometry, applying Beer–Lambert’s law and resolving purity by the following calculation: (A260/A280) = (A260 reading − A320 reading)/(A280 reading − A320 reading).

Primer3 software (Version 4, Thermo Fisher Scientific, Walthan, MA, USA) was used to design exon-spanning primers that flanked introns preventing amplification of genomic DNA contaminants. Temperature gradients were used to verify the predicted optimal annealing temperature of 60 °C in a thermocycler with REDTaq PCR Master Mix (Sigma-Aldrich, Oakville, ON, Canada) on pooled cDNA, and products were subsequently electrophoresed on 1% agarose gels.

SYBR^®^ Select Master Mix (Life Technologies, Thermo Fisher Scientific, Walthan, MA, USA, Canada) was used, as per the manufacturer’s instructions, and the reactions run on a 7900HT qPCR thermocycler (Applied Biosystems, Thermo Fisher Scientific, Walthan, MA, USA). Software analytics in SDS v2.4 (Applied Biosystems, Thermo Fisher Scientific, Walthan, MA, USA) performed _∆∆_Ct computations (as-plotted). Briefly, results were normalized to the expression of the housekeeping gene *glyceraldehyde 3-phosphate dehydrogenase* (*GAPDH*) and expression was relative to initial time points (time = day 1).

Primers (*Mus musculus*) included: *tissue non-specific alkaline phosphatase (Tnap)* amplifying 117 bp: (+) 5’-GGGAGATGGTATGGGCGTCT-3’, (−) 5’-AGGGCCACAAAGGGGAATTT-3’; *osteonectin* (*On*) amplifying 119 bp: (+) 5’-CCCATGGAACATTGCACCAC-3’, (−) 5’-TCCTTGTTGATGTCCTGCTCC-3’; *SP7* amplifying 151 bp: (+) 5’-TCTCTGCTTGAGGAAGAAGCTC-3’, (−) 5’-GGGCTGAAAGGTCAGCGTAT-3’; and *GAPDH* amplifying 111 bp: (+) 5’-AAGGGCTCATGACCACAGTC-3’, (−) 5’-CAGGGATGATGTTCTGGGCA-3’. *M. musculus* primers were confirmed to not anneal/cross-amplify with transcripts from *H. sapiens*. To this end, transcript markers for osteoblastic differentiation of MC3T3-E1 cells were verified with pooled 1833-TR transcripts.

### 2.4. Analysis of Construct Mineralization

#### 2.4.1. Attenuated Total Reflectance-Fourier Transform Infrared Spectroscopy (ATR-FTIR) and Microscopy

ATR-FTIR spectroscopy was carried out using a Spectrum 400 (PerkinElmer, Walthan, MA, USA). Constructs were washed with molecular biology-grade water (3 × 15 min), then lyophilized in a Benchtop K series freeze dryer (VirTis, Gardiner, NY, USA) for 16 h at −103.2 °C and 11 mTorr. Analysis was performed at a resolution of 16 cm^−1^ with a total of 64 scans. The Fourier transformation algorithm was introduced by Spectrum (software) v.6.3.1 (2007) (PerkinElmer, Walthan, MA, USA). Incident radiation detected bond energies within mid-range infrared (650–2500 cm^−1^) and spectra were normalized to organic amide I in the 1680 to 1700 cm^−1^ footprint region for inter-chain amide-carbonyl bonds present in type I collagen.

For FTIR microscopy, a Spotlight 400 (PerkinElmer, Walthan, MA, USA) was attached to the ATR-FTIR apparatus to permit topographic assessment. The field of view was set to 100 µm by 200 µm of the sample, with a point size of 1.56 × 1.56 µm interval of −8.0 cm^−1^, and a resolution of 16 cm^−1^ over 16 scans per sampled point. The interferometer speed was set to 1 cm/s.

#### 2.4.2. X-Ray Diffraction (XRD)

XRD (Bruker D8 Discovery Series, Milton, ON, Canada) was used to characterize the presence of mineral in the DC gels. Samples were prepared as described for ATR-FTIR. Detection of X-radiation occurred between 10° and 80° two-theta with a 500 µm sample (planar) oscillation. Diffractograms were cross-referenced with the International Centre for Diffraction Data (ICDD) database.

#### 2.4.3. Construct Reaction with Silver Nitrate

Constructs were washed (3 × 15 min) in molecular biology-grade water prior to incubation in a 5% sodium nitrate solution for 120 min. Constructs were placed directly under a compact fluorescent lap (CFL) bulb at 1200 lumens at 6500 K (color temperature) for 45 min, then washed in molecular biology grade water for 3 × 5 min. Samples were incubated and agitated in sodium thiosulfate (3 × 5 min washes), then washed in molecular biology grade water (3 × 15 min). The resulting analytes were visualized by optical (light) microscopy (40×).

### 2.5. Statistical Analysis

All data were statistically compared with one-way analysis of variance (ANOVA) tests.

## 3. Results

### 3.1. Matrix Mineralization by MC3T3-E1 Pre-Osteoblast Cells

**Figure 1:**
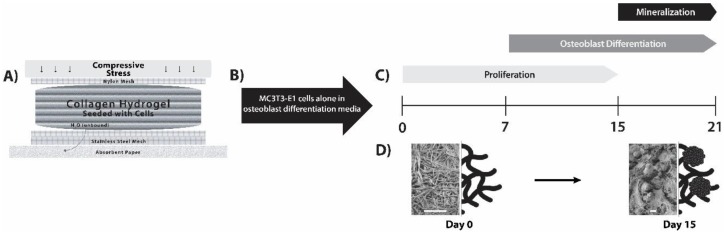
MC3T3-E1 differentiation and matrix mineralization in a dense collagen (DC) hydrogel. (**A**) Fabrication of DC hydrogels was carried out by plastic compression. The apparatus used to obtain plastic compression is diagrammatically displayed (adapted from [14]). (**B**) MC3T3-E1 cells were pre-seeded into the collagen hydrogels, and β-glycerophosphate and ascorbic acid were added to provide an osteoblastic differentiation medium. (**C**) Cells proliferated within the scaffolds and differentiated into osteoblast-like cells. Mineralization of the collagen scaffold by these cells was apparent at day 15. (**D**) Using this DC gel model, an “osteoid-like” collagen matrix at day 0 was converted into mineralized collagen within the 3D cell culture model (images presented are scanning electron microscopy (SEM), where scale bars are 15 µm and to the right are schematic depictions for illustrative purposes; rosettes for day 15 indicate hydroxyapatite (HA)).

**Figure 2:**
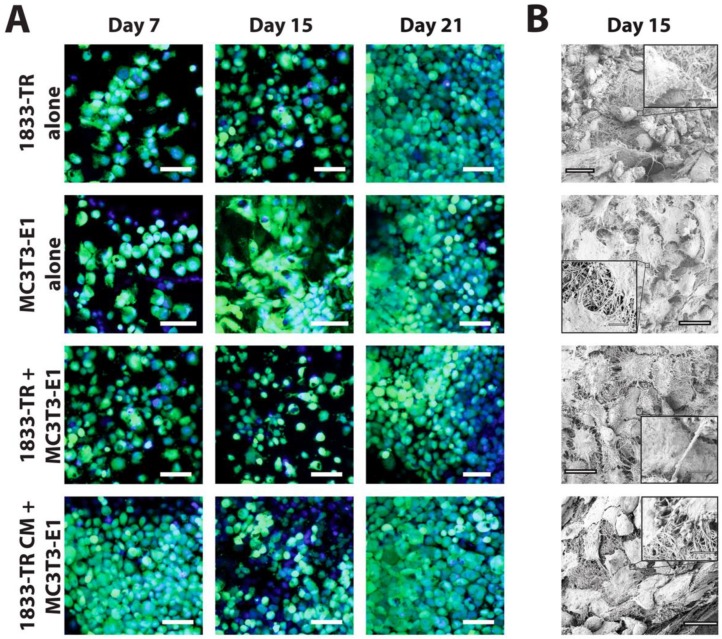
Cellularization of DC gels. (**A**) Cellularity of DC constructs visualized by confocal laser scanning microscopy (CLSM). Cell bodies (green, Calcein AM) and nuclei (blue, Hoechst 33342) were visualized at days 7, 15, and 21 in culture. DC constructs employing 1833-TR CM or co-culture approaches are indicated. Scale bars represent 55 µm for magnified images at 40×. (**B**) SEM micrographs at day 15 in culture depicting cuboidal (1833-TR) and spindle-like (MC3T3-E1) cellular morphologies. Scale bars represent 20 µm (large image) or 250 nm (image insets).

A DC gel system that enables seeding of pre-osteoblast cells in a 3D collagenous matrix, which permits subsequent mineralization of the scaffold under differentiation conditions, was employed (Figure 1). Gelification and subsequent plastic compression to generate DC constructs preserved cell viability up to day 21 in culture as assessed by active cytosolic esterases (Calcein AM staining) and intact nuclei (Hoechst 33342) using CLSM (Figure 2A and Figure S1A). The homogenous distribution of cells was observed in all DC constructs by day 7 post-seeding. The proliferation of cells was evident throughout the time course of the experiment, with saturation of high-powered fields with live cells occurring by day 21 post-seeding.

Surface characterization further confirmed construct cellularization by the use of SEM (Figure 2B and Figure S1B). CLSM revealed individually discernable cells at day 15 post-seeding; whereas SEM micrographs allowed for the characterization of cell morphology at this time point.

### 3.2. Construct Dynamism and Molecular Biology

**Figure 3:**
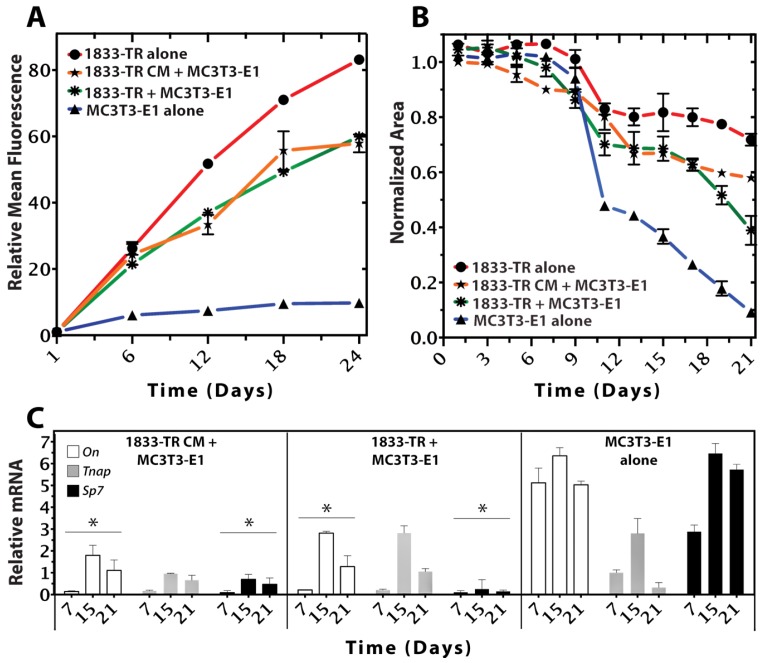
Cellular growth, survival, and differentiation within the 3D scaffold. (**A**) Resazurin-reduction (alamarBlue^®^) Assay (relative fluorescent intensity units) of cell-seeded constructs at days 1, 6, 12, 18, and 24 in culture. Metabolic activity of 1833-TR (circles), and to a lesser extent, co-cultures (stars) and 1833-TR CM (asterisks; with MC3T3-E1 cells present) increased with time in culture. In contrast, the metabolic activity of MC3T3-E1 cells alone (triangles) reached a plateau after day 12. Error bars indicate standard deviations of three independent experiments, each performed in triplicate per condition. (**B**) Cell-mediated gel contractility assays. Changes in relative surface area from the initial time point (day 0) to the subsequently indicated time points (days) are plotted. At day 12, an inflection point was observed in a cell-mediated gel-contractility assay for MC3T3-E1 alone, indicating that the construct was being remodeled by the cells to a greater extent than when 1833-TR cell or the medium that they conditioned was present. (**C**) RT-qPCR analyses of osteoblast differentiation markers (*On*, *Tnap*, *SP7*) normalized to *GAPDH*. The ratio of normalized gene expression for each marker (days 7, 15, 21) relative to the initial normalized expression values (day 0) are shown. Data revealed suppression of markers that indicate osteoblast differentiation with significance (* *p* < 0.05 On, Sp7; comparison to MC3T3-E1). Although alkaline phosphatase (*Tnap*) did not statistically decrease, a downwards trend by day 21 was evident.

Cell metabolic activity, measured by a commercial resazurin-reduction assay (alamarBlue^®^), indicated an increase in resorufin fluorescence over the course of 24 days in culture (Figure 3A and Figure S2A). DC gels seeded with 1833-TR cells alone showed the greatest increase in fluorescence over time, suggesting sustained proliferation. In contrast, gels containing only MC3T3-E1 cells revealed a plateau in fluorescence at day 6 post-seeding, suggesting diminished proliferation and enhanced osteoblast differentiation. MC3T3-E1 cell proliferation was shown to plateau by day 6, relative to day 1, in culture (*p* < 0.0001).

A cell-mediated gel-contraction assay was used as an indicator of the capacity for seeded cells to remodel the scaffold. There was a sigmoidal relationship between the construct surface area and time (Figure 3B and Figure S2B). DC gels seeded with only 1833-TR cells exhibited the least degree of contraction. In contrast, a significant degree of contraction was observed in constructs seeded with only MC3T3-E1 cells. The ability of MC3T3-E1 cells to remodel and contract collagen was significantly (*p* < 0.05) impaired in the presence of 1833-TR cells or CM-derived from 1833-TR cells.

qPCR analyses revealed changes in murine-specific gene transcript markers associated with MC3T3-E1 osteoblastic differentiation (Figure 3C). *SP7*, an early indicator of osteoblastic differentiation [30,31], was decreased in DC gels containing either 1833-TR derived CM and MC3T3-E1 cells, or 1833-TR/MC3T3-E1 co-cultures when compared to scaffolds containing only MC3T3-E1 cells. *Tnap* expression is a well-characterized marker of osteoblast differentiation, which increases early and persists to later stages of osteoblastic differentiation [32,33]. *Tnap* transcripts were shown to decrease when 1833-TR derived CM was combined with MC3T3-E1 cells, when compared to constructs containing only MC3T3-E1 cells. *On (SPARC)* is a gene encoding for a matricellular protein [34] that, when expressed, may be indicative of matrix remodeling in osteoblast cells. *On* expression decreased in DC gels containing either 1833-TR derived CM and MC3T3-E1 cells or 1833-TR/MC3T3-E1 co-cultures, when compared to scaffolds containing only MC3T3-E1 cells alone.

### 3.3. Construct Mineralization

ATR-FTIR spectroscopy indicated typical collagen peaks corresponding to amides I, II, and III in all constructs ~1650, ~1560, and ~1245 cm^−1^, respectively (Figure 3A and Figure S3A). There was a progressive increase in the *v*1 region of the phosphate peak at 1050 cm^−1^ in DC constructs seeded with MC3T3-E1 cells alone in response to osteogenic medium. Gels containing 1833-TR/MC3T3-E1 co-cultures or 1833-TR derived CM/MC3T3-E1 cells exhibited a significantly impaired peak this region.

**Figure 4:**
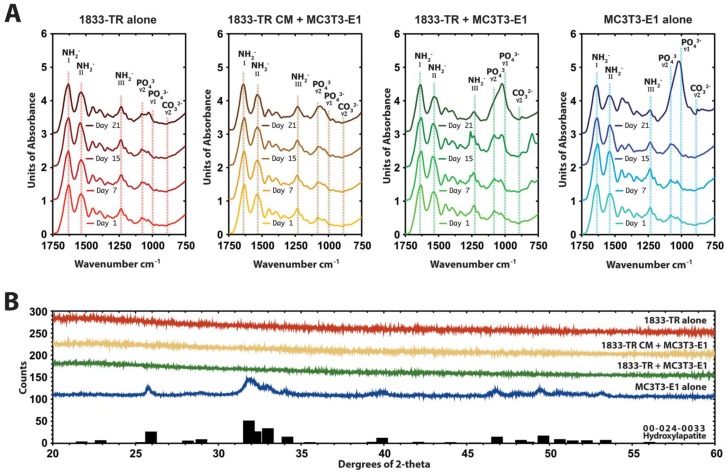
Mineral composition of DC gels. (**A**) Attenuated total reflectance-Fourier transform infrared (ATR-FTIR) spectroscopy of constructs at day 1, 7, 15, and 21 in culture**.** Characteristic absorption pattern peaks in the footprint regions are indicated. The amide I peak, which is centered at ~1650 cm^−1^ confirms the collagen triple helix. Bands between 1600 and 1500 cm^−1^ are attributed to amide II and the amide III peak can be identified at 1245 cm^−1^. At day 21 in culture, the shape of the phosphate peaks in the 1050 cm^−1^ region in MC3T3-E1 culture alone indicated HA formation. At this time point, there was a decrease in the presence of the phosphate peaks in co-cultures and 1833-TR CM + MC3T3-E1 constructs to MC3T3-E1 cultures. (**B**) X-ray diffraction (XRD) diffractograms of constructs at day 15. MC3T3-E1 seeded DC constructs displayed a crystalline structure compared to those seeded with 1833-TR cells alone, 1833-TR/MC3T3-E1 co-cultures and 1833-TR-derived CM with MC3T3-E1 cells at day 15. In particular, a broad peak at around 32 degree 2-theta suggested apatite formation.

XRD diffractograms of DC constructs seeded with MC3T3-E1 cells at day 15 in osteogenic medium revealed an 82% similarity to crystalline hydroxyapatite profiles (Figure 4B). In contrast, there was no detectable crystalline structure present in DC gels containing 1833-TR/MC3T3-E1 co-cultures or 1833-TR derived CM/MC3T3-E1 cells.

**Figure 5:**
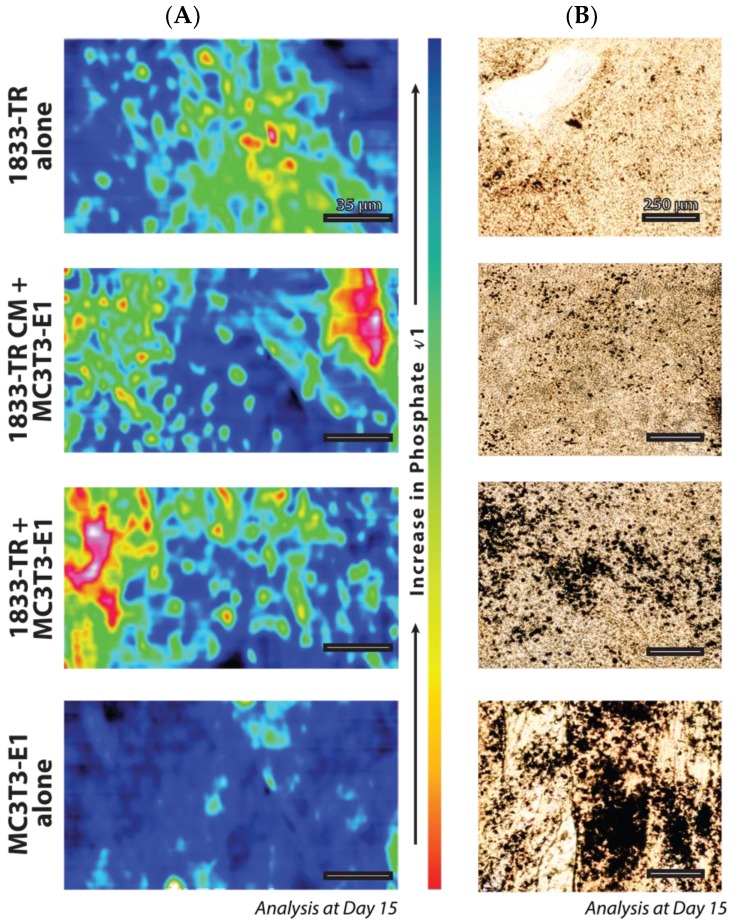
Geometric positional arrangement. (**A**) FTIR microscopy in a 200 × 100 µm field of view for the *v*1 phosphate peak at day 15 in culture. Order of *v*1 phosphate peak presence: MC3T3-E1 > CO-CULTURE > CM CULTURE > 1833-TR. The legend present for the increase in phosphate *v*1 indicates blue (more phosphate, more mineralized), and the opposite polar end, red (less phosphate, less mineralized). (**B**) Silver staining to detect areas of mineralization.

FTIR microscopy, which measures the positional arrangement of phosphate in the collagen constructs, revealed a more dispersed pattern with 1833-TR derived cell-CM/MC3T3-E1 cells when compared to 1833-TR/MC3T3-E1 co-cultures (Figure 5A). Whole construct histological analysis revealed the presence and location of phosphate species in a topographical manner (Figure 5B and Figure S3B). Nitrate displacement, which yielded silver phosphate (von Kossa) deposits, revealed a more dispersed pattern of mineral in collagen constructs harboring 1833-TR derived CM, plus MC3T3-E1 cells when compared to localized foci of mineral in scaffolds containing 1833-TR/MC3T3-E1 co-cultures at day 15.

## 4. Discussion

The work presented here describes a plastically compressed, DC hydrogel system as an in vitro 3D model to examine the effects of either breast cancer CM, or a breast cancer cell co-culture, on osteoblast-mediated matrix mineralization. Previous attempts to model such interactions in a 3D environments have suggested the utility of such scaffolds in therapeutic testing [35,36,37,38,39,40,41,42,43], with the growing realization that anti-cancer drug responses may be modulated by cell–matrix interactions. Attention has also been drawn to the utility of such in vitro models to mechanistically understand how disseminated tumor cells interact with elements of the stroma [44,45,46,47,48], and notably, how tumor cell infiltration affects osteoblast-mediated mineralization. In one such study, investigators found that highly hydrated (HH) type I collagen hydrogels provided a sufficient platform for solid tumor development using MDA-MB-231 cells [49]. Cell necrosis and vascularization/angiogenesis (VA) markers were found to be up-regulated in cancer cells at a depth specifically between 150 µm and 200 µm within the gels. This provided mechanistic data, revealing the minimal depth for expression of VA markers. A shortcoming attributed to HH gels is their inability to allow for the long-term expansion of cancer cells in vitro (e.g., ≤7 days). In contrast to HH gels, over 90% of unbound water is removed during the fabrication of plastically compressed DC gels [14]. Less water results in a greater probability for physical interactions between collagen fibers, which strengthens the gel ultrastructure (reviewed in [50]) providing mechanically stiffer compressed gels [51]. It is due to this property that MC3T3-E1 cells are able to remodel the gel while preserving ultrastructural characteristics of the matrix; albeit with reduced surface area. A failure to actively remodel the matrix, which is indicated by the lack of surface area diminishment, may suggest a block in osteoblast differentiation. Thus, in conjunction with PCR revealing a diminished transcription level of osteoblasts markers relative to the osteoblast control, the results in this study imply that TNBC cells negatively affect osteoblast differentiation by evidence through the reduced surface area that is observed in the gel contraction assay.

While TNBCs are broadly metastatic, approximately 20–40% of the patients develop bone metastases [52,53], often resulting in shorter survival times when compared with patients who suffer from ER+ breast cancer [54]. In this regard, the MDA-MB-231 model, and its derived sublines, is the most routinely used in vivo model of osteolytic bone metastasis from breast cancer [55,56,57,58,59,60,61]. To this end, this aggressive breast cancer model was used in the 3D compressed DC hydrogel system to study breast cancer cell metastasis to bone, in vitro. In fact, the observation that TNBC cells inhibit osteoblast-mediated mineralization has been previously described [62]. This observation is in agreement with an independent study [63] that developed a bioreactor to gain insights into TNBC-mediated suppression of osteoblast differentiation [64]. The latter study was adapted from a previously described system [65] that possessed significant advantages, such as a constant supply of fresh medium, permitting long-term culture (e.g., >40 days using a bioreactor setup). Models such as this make use of degradable polyesters such as poly(lactide-co-glycolide) [24], Surlyn^®^ 1702 (an ionomer of ethylene acid copolymers) [22], alginate [23], and chitosan [20,21]. While useful in determining cell–cell interactions that might alter treatment response, these models do not fully recapitulate cell–mineral or cell–mineral–matrix interactions, due to the fact that they are not native extracellular matrix (ECM) substrates found in bone. Type I collagen is a native stromal ECM component in bone, with controllable properties [14,18,25,66,67,68], justifying use of the model that we have employed in this study.

The endosteal niche within the bone is vascularized, and it is rich in hematopoietic stem cells (HSCs), osteoprogenitors, and osteoblasts among other cell types. Chemokines and soluble factors attract disseminated tumor cells to the endosteal niche, as they bear similar requirements to thrive as HSCs [69]. These include the presence of Wnt proteins, TGF-β and CXCL-12 [70]. In competition with HSCs, tumor cells, may lay quiescent once they have gained access to this niche from the vasculature. A principle component of the endosteal niche is the osteoblast, which may support early tumor cell survival after seeding the bone marrow [71]. The model proposed here may provide a tractable system to allow an in-depth analysis of tumor cell osteoblast interactions that occur in a 3D, collagen-rich microenvironment.

MC3T3-E1 pre-osteoblast differentiation and mineralization by medium supplementation with ascorbic acid and β-glycerophosphate has been well characterized [72]. It was shown in this paper that mineralization was apparent at day 15. When MC3T3-E1 cells were cultured in conjunction with 1833 cells, or media 1833 cells conditioned, mineralization was impaired. This phenomenon is well in accord with other work, which attests that decreases in mineralization are not solely a product of osteoclast-mediated resorption [73]. One such investigation described nephroblastoma overexpressed protein (NOV/CCN3, reviewed in [74]) as a matricellular factor that is highly expressed in aggressive bone metastases produced by tumor cells [75]. It was further found that in 2D culture assays CCN3 impaired osteoblast differentiation and promoted osteoclastogenesis. Moreover, recombinant CCN3 achieved similar results when incubated with primary bone marrow cultures, revealing a decrease in alkaline phosphatase with the increases in CCN3.

Though a putative tumor-derived factor may prevent osteoblast differentiation and matrix mineralization, subtle differences in the pattern of mineralization was observed between breast cancer/osteoblast co-cultures when compared to MC3T3-E1 cells incubated with breast cancer-derived CM. This was evident in both staining and infrared microscopy. In co-cultures, it is possible that local regions that show impaired mineralization coincide with pockets of breast cancer cells. This would result in the increased local concentration of tumor-derived factors that impair osteoblast-mediated mineralization. In contrast, inclusion of condition media containing breast cancer–derived factors would result in a more homogenous delivery of factors that can impair osteoblast differentiation, resulting in a more uniform reduction of mineralization across the scaffold. This was apparent in the diffuse pattern of mineralization that characterized scaffolds incubated in the presence of breast cancer-derived CM.

The development of methods to mineralize DC scaffolds has been an active area of research [66,76,77,78]. In fact, a geometrically aligned collagen scaffold resulted in enhanced osteoblast differentiation [18]. Given that work presented here suggests that TNBC cells infiltrate and interact with pre-osteoblasts/osteoblasts, it would be of interest to examine the effects of metastatic breast cancer cells (both blastic and lytic models) on the alignment of underlying collagen, and the impacts on osteoblast-mediated mineralization.

## 5. Conclusions

This work demonstrates that a highly metastatic to bone subpopulation of MDA-MB-231 cells (1833 cells) impair MC3T3-E1 pre-osteoblast differentiation to osteoblasts. This occurs both in response to treatment directly with 1833 cells, or with a medium conditioned by 1833 cells. In a normal course of differentiation stimulated with a osteoblast differentiation medium, MC3T3-E1 cells express osteoblast transcription markers and mineralize matrix by day 15. Interestingly, an arrangement of mineral, which differed between direct 1833 cell treatment of MC3T3-E1 cells and CM treatment of MC3T3-E1 cells, suggested that a configuration of tumor cells forms. This chain-like structure, known as a single-file formation suggests potential for further investigation, both in a model setting, but also to investigate the clinical relevance of targeting contractile apparatuses.

## Supplementary Materials

The following are available online at http://www.mdpi.com/2079-4983/9/4/72/s1, Figure S1: Cellularization of DC gels, Figure S2. Cellular growth and survival, Figure S3. Mineral analysis of basal medium constructs.
