# Supplementary material for: A Three-Dimensional Dense Collagen Hydrogel to Model Cancer Cell/Osteoblast Interactions
Source: J Funct Biomater. 2018 Dec 12;9(4):72. doi: 10.3390/jfb9040072 (PMC6306762; doi:10.3390/jfb9040072)
Supplement: Supplementary file 1 [file jfb-09-00072-s001.pdf]

Supplementary Figures

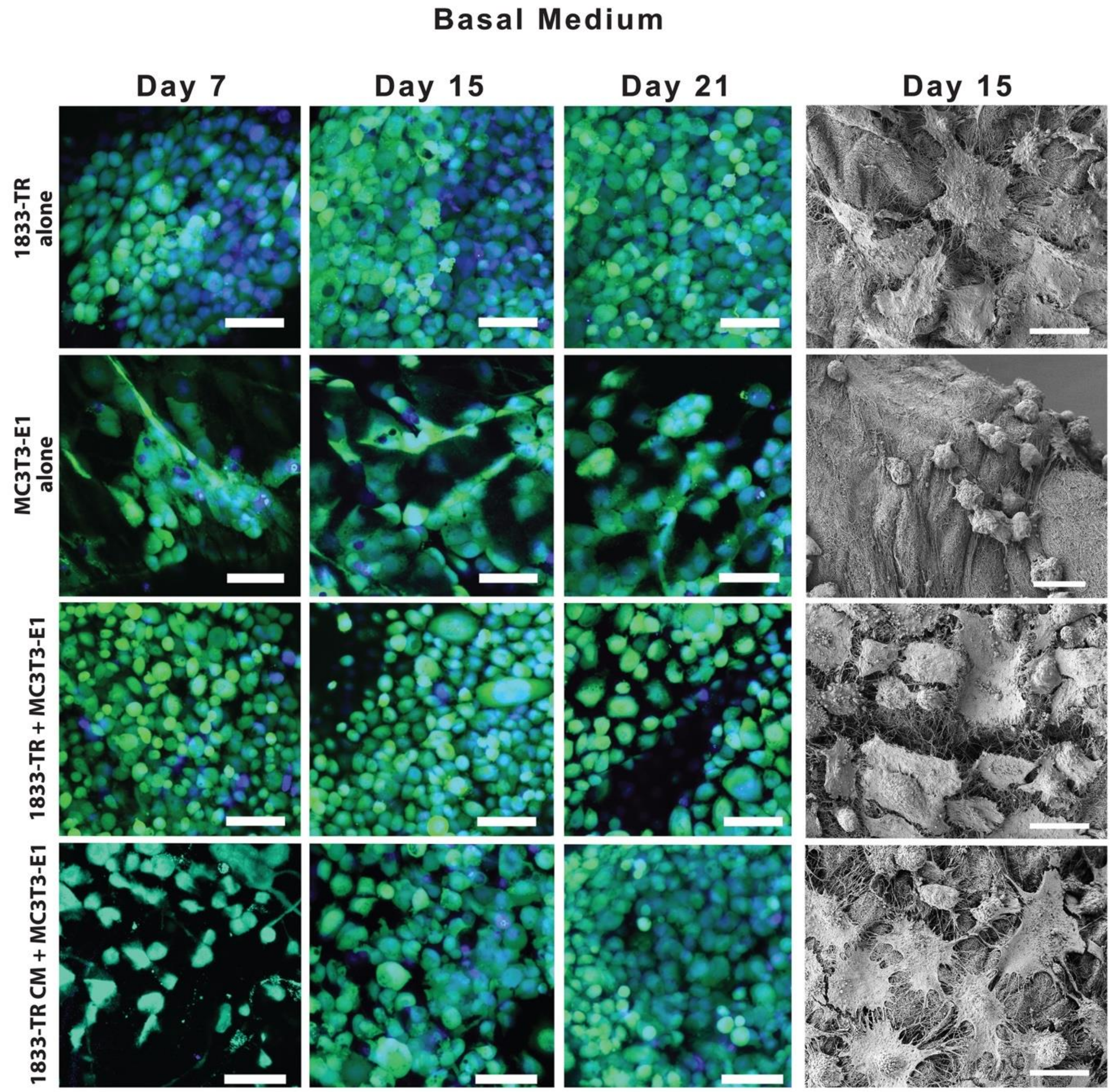

Figure S1. Cellularization of DC gels. (A) Cellularity of constructs visualized by CLSM in basal cell culture medium. Cell bodies (green, Calcein AM) and nuclei (blue, Hoechst 33342) were visualized at days 7, 15 and 21 in culture. The use of CM and co-cultures are indicated. Scale bars represent 55 µm for magnified images at 40x. (B) SEM micrographs are shown for day 15 in culture. Scale bars represent 20 µm (large images) and 250nm (insets).

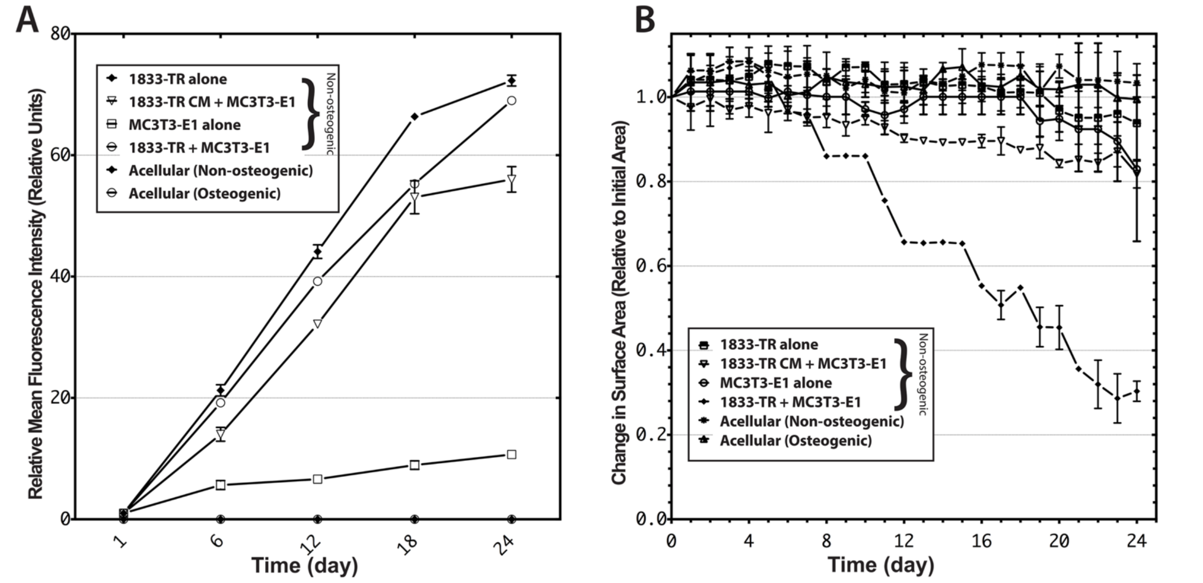

Figure S2. Cellular growth and survival. (A) Resazurin-reduction assay (alamarBlue®, relative fluorescence intensity units) of cell-seeded compressed DC constructs at days 1, 6, 12, 18, and 24 in culture. Error bars indicate standard deviations of 3 independent experiments performed in triplicate per condition. (B) Cell-mediated gel contractility assay. Relative changes in surface area of DC gels, relative to the initial time point, are plotted for the indicated time points (in days).

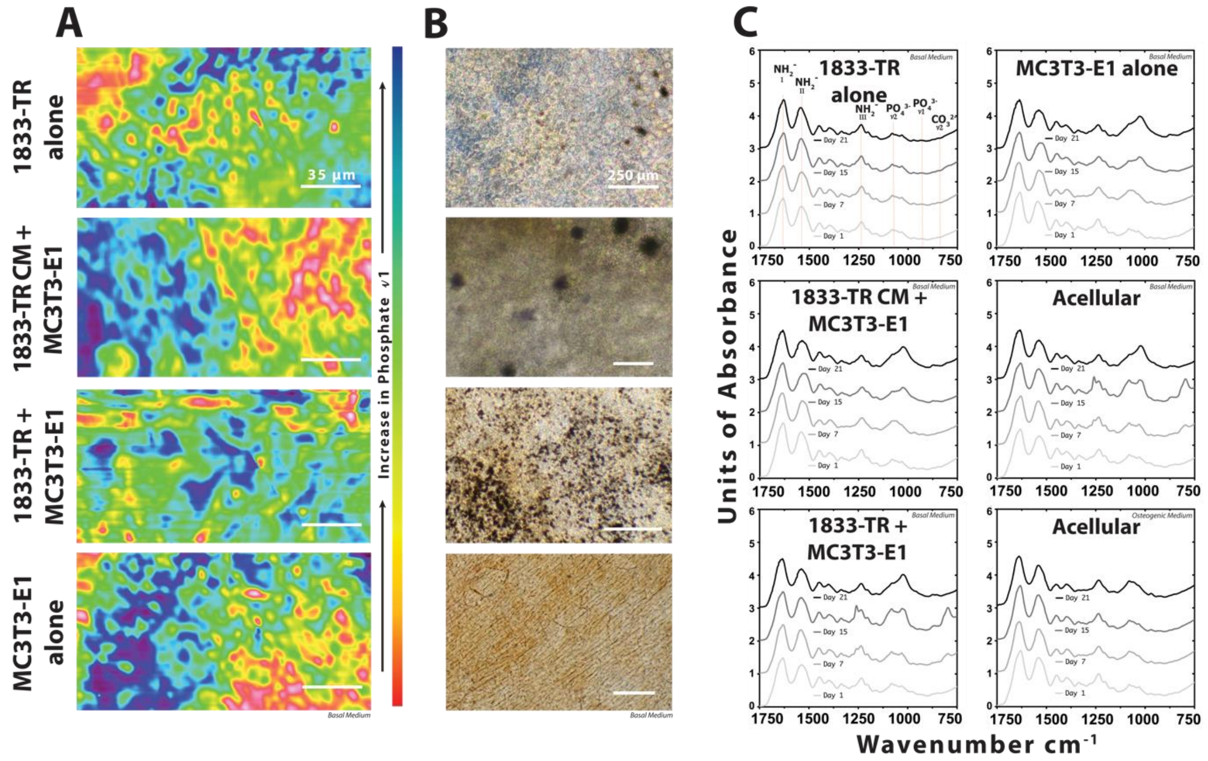

Figure S3. Mineral analysis of basal medium constructs. (A) FTIR microscopy in a 200 x 100 µm field of view for the *v*1 phosphate peak at day 15 in culture. (B) Silver staining to detect areas of mineralization. (C) ATR-FTIR spectroscopy of DC constructs at days 1, 7, 15 and 21 in culture. Characteristic absorption pattern peaks are indicated.
